# Discordant Manifestation of Congenital Heart Block in a Twin Pregnancy: A Case Report

**DOI:** 10.3390/diagnostics16172846

**Published:** 2026-09-04

**Authors:** Kristóf Levente Korpás, Olga Török, Tamás Deli, Lívia Beke, Balázs Kovács-Pászthy, Ágnes Horváth, László Orosz, Gábor Méhes

**Affiliations:** 1 Department of Pathology, University of Debrecen, 4032 Debrecen, Hungary; 2 Department of Obstetrics and Gynecology, University of Debrecen, 4032 Debrecen, Hungary; 3 Department of Pediatrics, University of Debrecen, 4032 Debrecen, Hungary; 4 Department of Rheumatology, University of Debrecen, 4032 Debrecen, Hungary

**Keywords:** autoimmune, congenital heart block, twin, histology, fibrosis

## Abstract

**Background**: Congenital heart block (CHB) is a severe disorder, with an estimated incidence of 1 in 20,000 live births. In most cases, the underlying pathophysiology involves transplacental transfer of maternal anti-Ro/SSA and/or anti-La/SSB antibodies, which are thought to bind to L-type calcium channels on fetal cardiac conduction cells, triggering apoptosis and initiating an inflammatory response that ultimately leads to destruction of the conduction system. **Case presentation**: We report a case of a dichorionic diamniotic twin pregnancy in which one fetus exhibited complete heart block in the 21st week of gestation, whereas the co-twin showed no abnormalities. The maternal history was notable for multiple autoimmune disorders among first- and second-degree relatives. Serological testing revealed markedly elevated titers of anti-SSA autoantibodies. Following parental decision, the pregnancy was terminated. Macroscopic evaluation of the affected fetal heart showed ventricular dilation, while histopathological examination demonstrated fibrotic and degenerative changes as well as focal calcification in the atrioventricular junctional region. In contrast, the cardiac conduction system of the unaffected fetus was preserved. **Discussion**: Fetuses of seropositive mothers have a 2–5% first-event risk of developing CHB during gestation. The available literature suggests that the underlying pathophysiology in discordant twin pregnancies likely reflects unequal placental transfer of antibodies in combination with subtle differences in fetal susceptibility and local immune responses. **Conclusions**: This case illustrates the unpredictable course of anti-SSA-associated CHB in twin pregnancies and highlights the importance of careful fetal cardiac surveillance in seropositive women, even in the absence of a clinically apparent autoimmune disease. Furthermore, the comprehensive histopathological documentation provides additional insight into the pathological basis of CHB.

## 1. Introduction

Congenital heart block (CHB) is among the most serious fetal cardiological conditions, affecting one in approximately 20,000 infants [1]. Affected fetuses, if born alive, face a myriad of complications, regardless of the underlying pathomechanism. In addition to structural heart diseases and very rare cases of inherited arrhythmia syndromes, autoimmune etiology encompasses a large fraction of cases (Table 1). Although the crucial events and mechanisms behind the pathogenesis of autoimmune CHB are now understood, the topic of CHB remains slightly controversial in its diagnostic, preventative and therapeutic aspects.

Histopathological studies performed on affected myocardial tissues universally reported a very similar appearance: the presence of fibrosis, calcification and chronic inflammatory infiltration consisting mainly of lymphocytes, macrophages and multinucleated giant cells in the location of the atrioventricular (AV) node. In many cases, endocardial fibroelastosis was also detected [2,3,4,5].

Anti-Ro/SSA antibodies are thought to play an initiating role in the pathogenesis of the rather specific myocardial involvement. These autoantibodies are directed against a ribonucleoprotein composed of two peptides, named Ro-52 and Ro-60, based on their molecular weight. The central segment of the Ro-52 peptide is termed p200 and evidence suggests that the presence of anti-p200 autoantibodies conveys the highest cardiac risk to the fetus [6,7,8]. During physiological fetal cardiac development, apoptosis takes place to sculpt and fine-tune the myocardial contractive and conductive elements. Preexisting autoantibodies then bind to exposed self-antigens, forming immune complexes and eliciting an inflammatory response. Besides that, molecular mimicry serves another possible role in pathogenesis. It is known that anti-SSA and associated antibodies can cross-react L-type calcium channels on conductive cardiomyocytes, likely causing disturbances in cellular calcium homeostasis [9,10]. The time window when fetal cardiac tissue is most susceptible to injury is the 18th–24th week of gestation; this reflects the time of myocardial tissue remodeling and maturation as well as the onset of placental immunoglobulin transfer [11].

Our aim was to expand the literature on CHB by reporting a rare case with clinicopathological correlations and thorough histopathological documentation.

## 2. Materials and Methods

Clinical data

Results of laboratory and physical examinations and imaging studies were extracted from the local hospital information system (UDMed 6.4.51, University of Debrecen, Debrecen, Hungary).

Tissue processing

Specimens from the Department of Gynecology and Obstetrics were received in a 4% phosphate-buffered formaldehyde solution (Sigma-Aldrich. St. Louis, MO, USA) and fixed for 36 h before autopsy, macroscopic examination and grossing. During the autopsy, no traditional anatomical dissection was performed on the fetal hearts. Both fetal hearts were sectioned into 6 identical sections in the horizontal plane. Based on the anatomical topography, the second section was expected to contain the AV junction area. After formalin-fixed paraffin-embedded processing, 2 μm thick slides were made. Besides routine hematoxylin and eosin (H&E) staining, additional Masson’s trichrome, elastica van Gieson stains and von Kossa impregnation were performed to better assess fibrosis and calcification. During the histopathological evaluation, identical sections from the fetal hearts were examined and compared.

For characterization of the inflammatory infiltrate and cellular environment, a panel of immunohistochemical (IHC) stains was performed: CD3, CD4, CD8, CD68 and CD163. We later expanded the panel to include SMA and CD71. Details of these IHC stains are displayed in Table 2. Appropriate positive controls were used to adjust the ICH protocols.

Slides were subsequently digitized using Pannoramic 480 Digital Scanner (3DHistech Kft., Budapest, Hungary) and microphotographs were taken using the SlideMaster 3.0.0 software (3DHistech Kft., Budapest, Hungary).

## 3. Case Presentation

Clinical history, follow-up

We report a 31-year-old multiparous (gravida 4 para 1) woman with a dichorionic diamniotic twin pregnancy. Her obstetrical history was positive for multiple early-stage spontaneous abortions in previous pregnancies. Otherwise, her medical history was unremarkable. In addition, multiple first- and second-degree relatives suffered from various autoimmune conditions (e.g., Hashimoto thyroiditis, Graves’ disease, uveitis, necrobiosis lipoidica, etc.).

**Figure 1:**
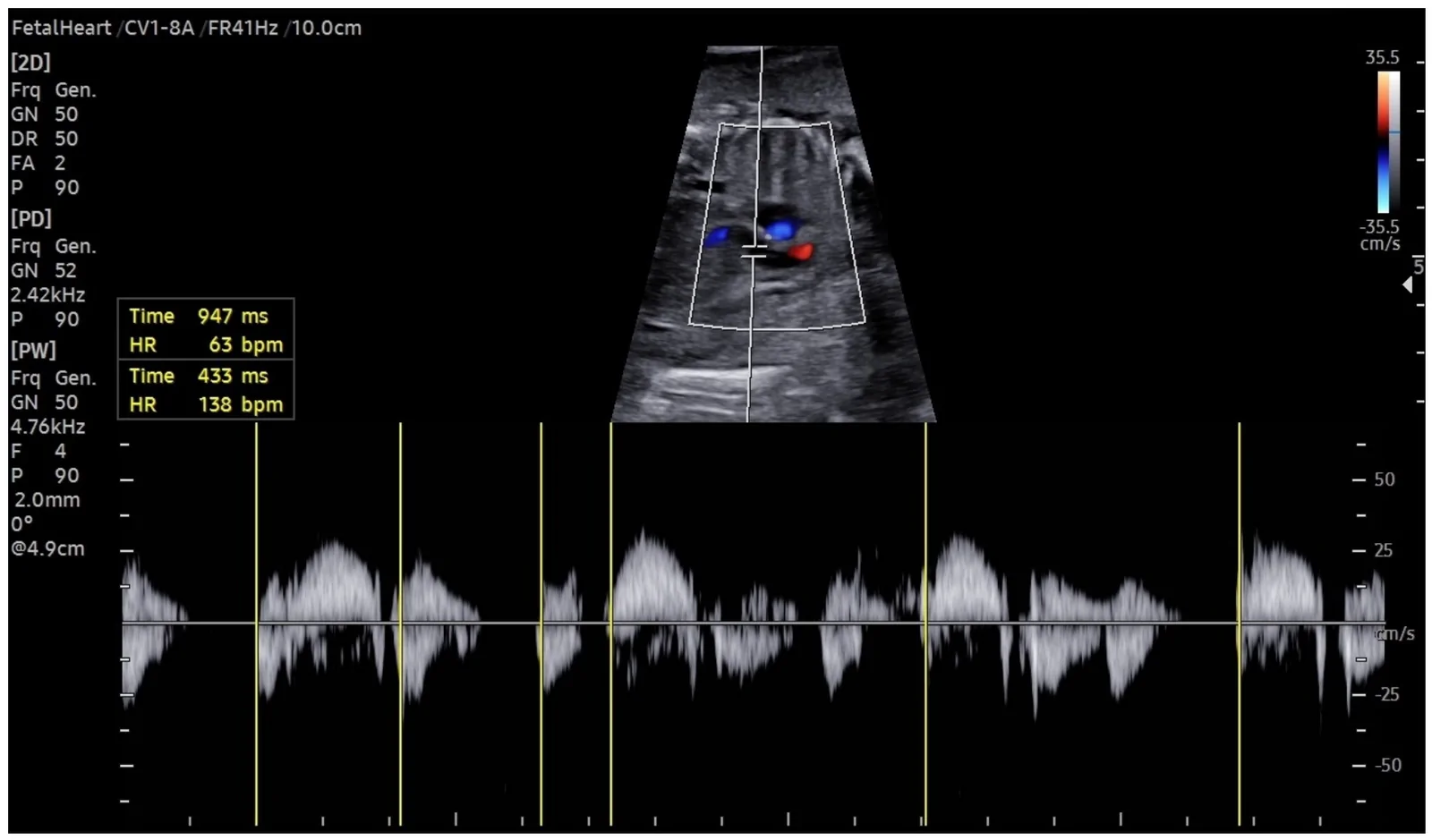
Representative echocardiographic image of the affected fetus, demonstrating the difference between atrial (138 bpm) and ventricular (63 bpm) pacing.

The pregnancy was uneventful and no adverse signs or symptoms were reported on either the maternal or fetal side. On the 21st week of gestation, the routine ultrasonographic examination revealed complete AV dissociation (third-degree AV block; complete heart block) of one fetus (fetus “A”) with the atrial rate being 138 bpm and the ventricular rate being 63 bpm (Figure 1). The heart was otherwise structurally normal. The co-twin (fetus “B”) showed no cardiac conduction abnormalities.

Subsequent maternal serological testing revealed markedly elevated anti-SSA levels and antinuclear antibody (ANA) positivity with a speckled nuclear pattern. Anti-dsDNA, anti-beta-2 glycoprotein I (B2GPI) and anti-phosphatidylserine antibodies, lupus anticoagulant and cardiolipin were negative. Complement C3 level and thyroid function was within the expected range. Detailed immune serology results are listed in Table 3.

Following genetic counseling, the parents decided to terminate the pregnancy at the 23rd week of gestation. Given the obvious immunopathological background and parental decision, no additional clinical genetic analysis was indicated. At the 6-month follow-up visit, the mother had developed Raynaud phenomenon and ophthalmic examinations had been performed because of new-onset eye dryness. Tear break-up time test was physiological (10 s); however, the Schirmer test indicated slightly reduced tear production (10–15 mm/5 min). Based on the clinico-serological findings, the diagnosis of undifferentiated connective tissue disease (UCTD) was made. At the time of writing this manuscript, no additional symptoms have been reported. The future treatment plan includes hydroxychloroquine (HCQ) and intravenous immunoglobulin (IVIG) administration at the time of potential future pregnancy to prevent the development of fetal CHB.

Autopsy and histopathological findings

The two placentas were fused; no superficial or deep vascular anastomoses were identified by thorough macroscopic examination. The dividing membrane structure was consistent with dichorionic diamniotic setting. Macroscopically, the two placentas—marked by vascular territories—appeared normal. Placenta “A” was 126 g, placenta “B” was 142 g; both had paracentral umbilical cords, and no focal or diffuse lesions were noted.

**Figure 2:**
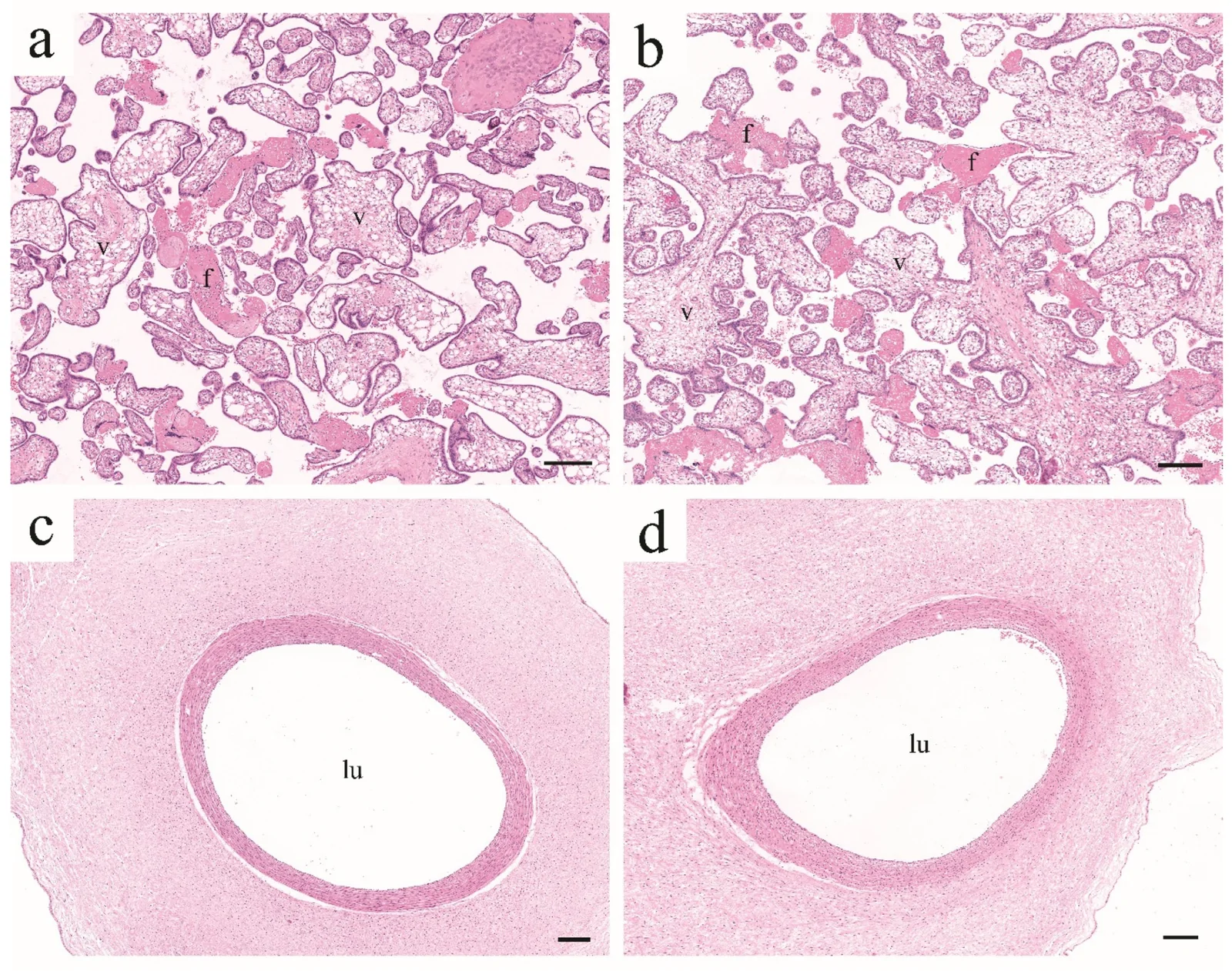
Histopathological findings of the placentas and umbilical cords. Villous edema and multifocal fibrin deposition were observed in the placental parenchyma of fetus “A” (**a**) and “B” (**b**). The umbilical cords of fetus “A” (**c**) and “B” (**d**) were preserved without significant histopathological abnormalities. H&E; scale bar = 200 µm; f: fibrin, lu: umbilical vein lumen, v: villus.

Microscopic examination of both placentas revealed marked edema and congestion in the villous compartment with multifocal fibrin deposition in the intervillous spaces (Figure 2). Placental arteries and arterioles exhibited mild irregular stenosis (up to 20% of the lumen cross-sectional area), but no vasculitis was detected (Figure S3d). In addition, there were no signs of parenchymal inflammation.

The umbilical cords were macroscopically and histologically normal. The chorioamniotic membranes were thickened by degenerative chorionic hyperplasia (Figure S1); the interstitial spaces were edematous and focal fibrinous exudate was seen (Figure 2).

Both fetuses were females and weighed an identical 486 g. According to the World Health Organization Fetal Growth Chart, this is consistent with 12th weight percentile, indicating physiological intrauterine growth rate [12]. There were minute variations in some of the other fetal biometrics among the twins, which are shown in Table 4. These differences are considered clinically insignificant, representing normal biological variation and possible postmortem measurement variability. Zygosity was undetermined.

Macroscopic external and internal examination revealed no developmental abnormalities; all the organs appeared normal for gestational age except for the heart of fetus “A”, which was slightly larger and exhibited dilated ventricles.

**Figure 3:**
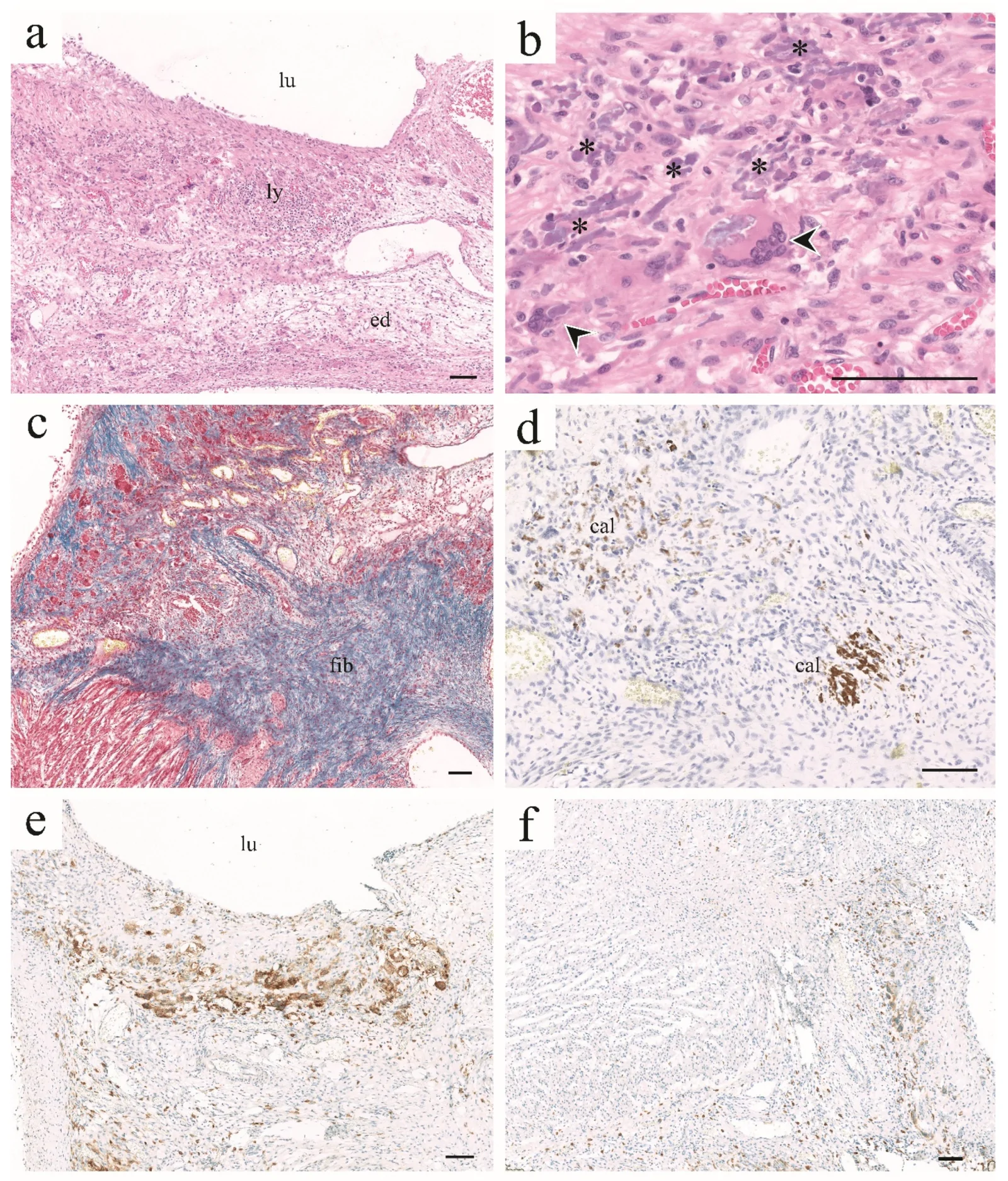
Histopathological findings of the AV junctional region of the affected fetus. Edematous fibrotic tissues with inflammatory infiltration composed of lymphocytes, macrophages and multinucleated giant cells are evident at low- (**a**) and high (**b**)-power magnification. Asterisks on (**b**) show focal calcification, arrowheads point at multinucleated giant cells engulfing calcified cellular debris, and H&E staining. Masson’s trichrome staining (**c**) emphasizes fibrosis, whereas von Kossa impregnation (**d**) highlights dystrophic calcification. CD4 (**e**) and CD163 (**f**) immunohistochemistry characterize T-lymphocytes and macrophages, respectively. Scale bar = 100 µm; cal: calcification; ed: edema; fib: fibrosis; lu: left ventricular lumen; ly: lymphocytes.

In both fetuses, pulmonary, renal and cerebral tissues were congested but otherwise normal for gestational age. Splenic and hepatic parenchyma was preserved; extramedullary hematopoiesis was observed in the liver, with hemophagocytosis in case of fetus “A” (Figure S2). The most contrasting differences were found in the cardiac tissues. Cardiomyocytes of fetus “B” were swollen, depleted, but otherwise oriented, structurally correct, and accompanied by focal mild subendocardial fibroelastosis (Figure S2l,m). Fetus “A” similarly displayed oriented but depleted and swollen myocardium (Figure S3a,b). These findings may represent mild antibody-associated injury, although their nonspecific nature limits interpretation. However, in the lower segments of the interatrial septum and the AV junction—where the AV node is located—an ill-defined, markedly fibrotic and edematous area was seen with prominent degenerative changes (Figure 3a–c). The accompanying inflammatory infiltrate was composed mostly of lymphocytes and activated macrophages with the presence of scattered multinucleated giant cells engulfing cellular debris (Figure 3e,f). Von Kossa staining highlighted many foci of granular dystrophic calcification (Figure 3d). At the periphery of the lesion, a rim of degenerating cardiomyocytes could be seen. In addition, confluent endocardial fibroelastosis was also seen (Figure S3c).

## 4. Discussion

CHB is a severe cardiac complication of pregnancies in anti-SSA seropositive women with or without an established autoimmune disease. In a substantial proportion of cases, CHB is the first manifestation of underlying seropositivity in the absence of apparent clinical symptoms. Approximately half of these women later develop UCTD—as seen in our case—or a differentiated autoimmune disorder, whereas the other half remain asymptomatic [13]. The clinical management of anti-SSA-positive pregnancies varies among countries and clinical centers. Prenatal ultrasonography (US) is the standard diagnostic tool, where physicians can indirectly evaluate the state of the fetal conduction system. Second-degree and complete (third-degree) AV block is virtually evident upon fetal echocardiography; however, the diagnosis of first-degree block without an electrocardiogram is rather difficult. During US examination, the “mechanical” PR interval can be estimated via multiple approaches [14]. Based on the results, one can predict the subsequent development of higher-degree blocks. According to the guidelines published by the American College of Rheumatology, weekly serial fetal echocardiography monitoring between the 16 and 26th weeks of gestation is recommended for anti-SSA (and/or SSB)-positive pregnant women with a history of a prior infant with CHB. In case of negative maternal anamnesis, fetal cardiac US monitoring could be less frequent [15]. Despite the proposed weekly screening period, one study found that the sequence of first-degree to complete heart block can be shorter than a week, suggesting a need for even more frequent monitoring [16]. Discovering first-degree heart block, one of the first signs of potential underlying cardiac destruction, is essential for successful outcomes. Evidence suggests that conversion of second to first-degree block and first-degree block to sinus rhythm is possible with adequate pharmacological treatment [17].

In recent decades, fluorinated glucocorticoids were the first-line options for treating CHB, although, to date, no reports are available on the successful conversion of complete heart block, further highlighting the need to detect early signs. Similarly, no evidence has demonstrated improved survival with the use of beta sympathomimetics, which are often used when fetal heart rate is below 50–60 bpm [18]. Once complete heart block has developed in utero, the risk for fetal hydrops and neonatal left ventricular dysfunction increases. Knowing that CHB and the cellular damage behind that is permanent, the emphasis is on primary and secondary prevention. Recently, home monitoring was proposed as an approach for detecting the early signs of a possible heart block [18].

It is widely accepted among rheumatological and immunological professional societies that hydroxychloroquine (HCQ) should be administered for prevention of CHB in mothers with a previous affected pregnancy, since it was shown to significantly decrease the recurrence risk of developing CHB in subsequent pregnancies. Some guidelines recommend HCQ use for anti-SSA-positive pregnant women even without positive history. Fluorinated glucocorticoids remain the recommended drugs of choice in cases of first- and second-degree fetal heart block, but their use in complete heart block is questionable because of the uncertain benefits stated above [15,19,20,21]. In some clinical centers, treating physicians use intravenous immunoglobulin (IVIG) in seropositive mothers for prevention as well as treatment; however, results from multiple studies suggest the inefficacy of IVIG treatment in this setting [22,23]. After birth, the vast majority of neonates require pacemaker implantation, and while prognosis of these infants is generally excellent, approximately 2–6% of them eventually develop dilated cardiomyopathy and consequent left ventricular failure, representing a clinically significant morbidity associated with poorer overall outcome. For these patients, a heart transplant is the last-resort treatment option, which is a serious health burden [24,25]. Therefore, genetic counseling should provide the parents with evidence-based information about the expected long-term morbidity and mortality associated with CHB. In discordant twin pregnancies, it is a challenge to predict whether the unaffected co-twin remains asymptomatic or develops CHB through the remainder of the pregnancy. In our case, this uncertainty, paired with the anticipated lifelong disease burden, was the main reason underlying the parents’ decision to terminate the pregnancy.

The present case illustrates a twin pregnancy complicated with discordant CHB, an overall rare condition, where anti-SSA seropositivity was identified during pregnancy and the cardiac findings were consistent with autoimmune-mediated injury. The clinical course presented in our case report in the small group of affected patients with twin gestation is not as unusual as it first seems. According to a comprehensive review of the condition, in approximately 70% of anti-SSA-positive twin pregnancies, the manifestation of CHB is discordant [26]. The majority of the publications available on the topic of CHB feature singleton pregnancies. The first discordant twin study dates to 1985, when Harley et al. reported an anti-SSA-positive mother with twin pregnancy [27]. Since then, the number of published cases has increased to 26, of which 18 appeared in registry/cohort studies and eight in individual case reports, including this present paper [11,27,28,29,30,31,32,33,34,35,36,37]. The clinical outcome varied among these cases: one mother experienced intrauterine fetal demise (IUFD) in the 31st week of gestation, whereas six sets of twins survived after birth, and in our case, the parents decided to terminate the pregnancy because of potential IUFD and other complications. Of the six live-born fetuses with CHB, four underwent pacemaker implantation (three endo- and one epicardial), one received adrenergic agonists, and one needed no pharmacotherapeutic or surgical support because of sufficient heart function. However, long-term outcomes are not described in any of those cases.

Research data consistently shows that seropositivity alone is not sufficient for disease manifestation, since CHB develops only in approximately 2% of seropositive pregnancies [38,39]. It is well known, however, that a generally high antibody titer is required and pregnancies where the mothers have low autoantibody titers are considered low-risk for disease [40]. Because of the different methodical approaches research studies used to measure the level/titer of autoantibodies, comparison of the reported antibody titers across the literature is limited.

The mechanism behind fetal discordancy is yet to be understood; however, the literature proposes several contributing factors including variations in placental anatomy and perfusion, differential Fc receptor-mediated antibody transfer, fetal immune or genetic susceptibility and differences on the epigenetic level. In both dichorionic (DC) and monochorionic (MC) twin pregnancies, placental vasculature is not homogeneous, which can create marked differences in the intrauterine environment [41,42]. Thus, one twin might experience a greater exposure to maternal autoantibodies, than the other. Among the reported discordant twin CHB cases where chorionicity was described, nine out of ten pregnancies were DC, including our case, and only one was MC. It is well known that twins, even monozygotic ones, have phenotypical differences despite their identical genome. This can be explained by certain epigenetic modifications. Several studies showed that there are, indeed, variations in the DNA methylation pattern of certain tissues and cells of identical twins [43]. Translating this principle to the topic of CHB, a subtle difference in the DNA methylation pattern of fibrosis- or immune-related genes can manifest as normal function in one fetus but disease in the other; however, further genetic studies are needed to test this hypothesis. Some authors have suggested that chorionicity might influence epigenetic status and reported that MC twins exhibit higher variability than DC ones [44,45]. In contrast to that, as mentioned above, DC cases prevail in the reported group of twins discordant for CHB; one possible explanation behind the controversy can be the small case number of the cohort. Besides placental anatomy and epigenetic differences, placental Fc receptor-mediated antibody transfer and variable fetal immune susceptibility also need to be taken into consideration. It is suggested that neonatal Fc receptor (FcRn)-mediated antibody transfer has maternal, fetal and placental regulators, including fetal sex, placental Fc receptor expression or chorionicity [46]. Recent evidence showed that serum IgG levels positively correlate with the placental expression of FcRn [47]. Although direct evidence comparing FcRn expression between the placentas of dichorionic twins is lacking, differences in placental regulators of IgG transport may theoretically contribute to different fetal antibody exposure. Furthermore, in a 2012 study using a rat model of CHB, the authors found that major histocompatibility complex genes regulate susceptibility and play a role in fetal disease outcomes [48]. However, further studies including fetal autoantibody measurements are needed to explain the phenotypical differences; without this data, conclusions regarding differential transplacental autoantibody transfer remain speculative.

In recent years, in the field of translational laboratory research, type I interferons (IFN) were proposed as key cytokines orchestrating the destruction of affected myocardial tissues. Type I IFN upregulates the expression of Ro-52 and drives apoptosis. In addition, the serum levels of IFN-α were found elevated in both the mothers and fetuses of anti-SSA-positive pregnancies. Because of all that, the maternal IFN levels can be utilized as a potential biomarker of CHB [49,50].

It is worth noting that CHB can result from etiologies other than autoimmunity, such as structural heart diseases—either as part of a genetic syndrome or in isolation—viral myocarditis or inherited fetal arrhythmia syndromes. Fetal ultrasonography may help detect structural abnormalities, whereas channelopathies can only be diagnosed through prenatal or postnatal genetic testing. In our case, neither the initial ultrasonography nor the autopsy revealed any structural cardiac abnormalities. Histopathologically, the inflammatory infiltration and fibrosis were restricted to the AV junctional region, while no signs of potential myocarditis were observed elsewhere in the myocardium. As channelopathies are functional cardiac disorders that do not manifest with specific histopathological lesions, the observed inflammatory and fibrotic changes make a primary channelopathy less likely.

Taken together, pregnancies in anti-SSA-positive women may be complicated by CHB, which appears more likely to manifest discordantly in twin pregnancies, particularly in a DC setting. Although CHB is the result of a destructive histological process and hence permanent, it is preceded by potentially reversible stages; however, the time window of reversible to irreversible injury appears to be short. Secondary prevention by screening and adequate preventive pharmacotherapy can greatly decrease the risk. However, further investigation is needed to fully explore genetic, epigenetic, cellular and humoral elements of the pathogenesis and find potential biomarkers or targets for therapy.

Our case report expands the limited literature on discordant twin pregnancies complicated by CHB and provides high-resolution histopathological images demonstrating the cellular events in the cardiac conduction system of affected fetuses. Although histological images of cardiac tissue from human fetuses with CHB have been published, the majority date from the late 20th and early 21st centuries. We believe that the particular contribution of our case study lies in the comprehensive histopathological processing and imaging of the fetal AV junction.

As for limitations of the current case study, molecular genetic testing could have helped exclude inherited arrhythmia syndromes or other genetic conditions associated with cardiac conduction abnormalities, e.g., channelopathies or conditions with dilated cardiac ventricles, e.g., cardiomyopathies. Because of that, an additional conclusion arising from the present rationale is that, in the context of molecular testing, the potential coexistence of other genetically defined pathological factors should be considered in rare disorders.

In addition, fetal serological measurements could have provided information on antibody exposure and helped assess whether differential transplacental antibody transfer contributed to the discordant phenotype. Furthermore, determining zygosity would have strengthened the interpretation of discordance and the hypothesis of selective fetal susceptibility.

The histopathological evaluation was performed on postmortem tissue, and therefore, postmortem artifacts were unavoidable. In addition, there was a moderate discrepancy between the results of the initial ultrasound examination and the autopsy regarding ventricular dilation. This is partially attributable to the limitations of fetal ultrasound imaging; on the other hand, the assessment of ventricular dimensions at autopsy is inherently subject to some degree of methodological and interobserver variability because of the cardiac dissection technique. However, rapid progression to intrauterine heart failure and ventricular dilation cannot be excluded. Lastly, we acknowledge the limited ability of the terminated case to represent the natural history or long-term clinical course of congenital heart block.

Practical considerations:

The management of twin pregnancies complicated by maternal anti-SSA seropositivity remains challenging because of the inconsistent literature data. Guidelines are available for the management of women with established seropositivity, and evidence-based strategies exist for the prevention of recurrence. However, asymptomatic but seropositive women remain in a “gray zone”, as they are often diagnosed after complications have already developed. Although this present paper is neither a guideline, nor a comprehensive review, it offers the following considerations for clinical practice:

Since prevention is of utmost importance, a thorough medical history should be obtained from these women. If there are any findings suggestive of potential autoimmunity (positive family history, skin lesions, joint symptoms, previous abortions, etc.), the patient should be referred for basic immunological workup. If autoantibodies, including anti-SSA, are detected, and there are no contraindications, preventive treatment with HCQ and home monitoring of the fetal heart rate should be taken into consideration. For patients diagnosed only after fetal conduction abnormalities have already developed, corticosteroid therapy may be considered on an individual basis, particularly when a lesser degree of heart block is detected. These patients should also receive thorough genetic counseling.

Key findings:

This case report describes a dichorionic diamniotic twin pregnancy in which CHB developed discordantly in only one fetus and was diagnosed at 21 weeks of gestation. At the time of diagnosis, the mother had markedly elevated anti-SSA antibody levels but no clinical symptoms suggestive of an underlying autoimmune disease. Histological examination of the affected fetal heart revealed extensive fibrosis and degeneration, focal calcification, and inflammatory infiltration with multinucleated giant cells involving the AV junctional region, accompanied by endocardial fibroelastosis. During clinical follow-up, the mother was subsequently diagnosed with UCTD. In view of the recurrence risk in future pregnancies, a preventive treatment strategy including HCQ was planned.

## Figures and Tables

**Table 1:** Potential causes of CHB.

Structural heart diseases	Ebstein anomaly
	Left atrial isomerism (Heterotaxy syndrome)
	Corrected great vessel transposition
	Atrioventricular or interventricular septal defects
Inherited arrhythmia syndromes	Long QT syndrome
	Brugada syndrome (and other SCN5A-related syndromes)
	Progressive cardiac conduction disease
Anti-SSA positivity	Without systemic autoimmune disease
	With systemic autoimmune disease, e.g., systemic lupus erythematosus, Sjögren’s syndrome, etc.

SCN5A—sodium channel protein type 5 subunit alpha.

**Table 2:** Details of performed immunohistochemical staining.

Antibody							Antigen Retrieval		IHC Stainers	
Name	Vendor	Host	Cat. No.	Clone	Dil.	Incub. Time/Temp.	Method	Time/Temp.	Visualization System	Platform
CD3	Dako, an Agilent Technologies Company, Glostrup, Denmark	mouse	M725401-2	F7.2.28	1/100	30’/RT	HIER BERS1 pH 6	56’/100 °C;	Bond Polymer Refine Detection	Leica-Bond Max
CD68			M081401-2	Kp1	1/100	32’/RT	CC1 solution pH 8.5	20’/100 °C	Ultraview Universal DAB Detection Kit, 5269806001	VENTANA BenchMark ULTRA
SMA	Leica Biosystems Nussloch GmbH, Nussloch, Germany		M085101-2	1A4	1/1000	32’/RT	CC1 solution pH 8.5	20’/100 °C		
CD4	Roche (Hungary) Ltd., Budapest, Hungary		5552737001	SP35	RTU	16’/36 °C	CC1 solution pH 8.5	56’/100 °C		
CD8	Dako, an Agilent Technologies Company, Glostrup, Denmark		M710301-2	C8/144B	1/200	32’/RT	CC1 solution pH 8.5	56’/100 °C		
CD71	Merck KGaA, Darmstadt, Germany		171M-96	MRQ-48	1/200	16’/36 °C	CC1 solution pH 8.5	52’/100 °C		

BERS1—Bond Epitope Retrieval Solution 1; CC1—Cell Conditioning 1; DAB—diaminobenzidine; HIER—Heat-Induced Epitope Retrieval; IHC—immunohistochemistry; RT—room temperature; RTU—ready-to-use; SMA—smooth muscle actin.

**Table 3:** Immune serology results.

	Result	Reference Range
ANA pattern	fine/coarse speckled nuclear (AC-4.5)	-
ANA titer	1:1280	<1:80
anti-dsDNA antibody	1.8 IU/mL	0.0–20.0 IU/mL
anti-nucleosome antibody	2.00 U/mL	0.00–20.00 U/mL
anti-Smith antibody	<12 U/mL	0–12 U/mL
anti-Sm/RNP antibody	<12 U/mL	0–12 U/mL
anti-SSA antibody	>300 U/mL	0–12 U/mL
anti-SSB antibody	<12 U/mL	0–12 U/mL
anti-Scl-70 antibody	<12 U/mL	0–12 U/mL
anti-Jo-1 antibody	<12 U/mL	0–12 U/mL
anti-ribosomal-P-protein antibody	1.3 U/mL	<10.0 U/mL
anti-C1q antibody	2.60 U/mL	0.00–10.00 U/mL
anti-B2GPI IgG	18.2 U/mL	0.0–20.0 U/mL
anti-B2GPI IgM	<1.1 U/mL	0.0–20.0 U/mL
anti-B2GPI IgA	<4.0 U/mL	0.0–20.0 U/mL
anti-cardiolipin IgG	4.2 U/mL	0.0–20.0 U/mL
anti-cardiolipin IgM	2.0 U/mL	0.0–20.0 U/mL
anti-cardiolipin IgA	1.9 U/mL	0.0–20.0 U/mL

**Table 4:** Fetal biometrics.

	Fetus “A” (Affected)	Fetus “B” (Unaffected)
Weight	486 g	486 g
Crown–rump length	192 mm	215 mm
Crown–heel length	313 mm	309 mm
Toe-to-heel length	42 mm	41 mm
Head circumference	219 mm	206 mm
Thoracic circumference	173 mm	185 mm
Abdominal circumference	165 mm	141 mm

## Data Availability

The original contributions presented in this study are included in the article. Further inquiries can be directed to the corresponding author.

## Supplementary Materials

The following supporting information can be downloaded at https://www.mdpi.com/article/10.3390/diagnostics16172846/s1; Figure S1: Fetal membranes. Dividing membrane (a) was consistent with dichorionic diamniotic placentation. Chorioamniotic membranes of fetus ‘A’ (b) and fetus ‘B’ (c); H&E. Figure S2: Additional histological findings. Pulmonary parenchyma of fetus ‘A’ (a) and ‘B’ (b). Renal parenchyma of fetus ‘A’ (c) and ‘B’ (d). Cerebral parenchmya of fetus ‘A’ (e) and ‘B’ (f). Splenic parenchyma of fetus ‘A’ (g) and ‘B’ (h). Hepatic parenchyma of fetus ‘A’ (i) and ‘B’ (j). High power magnification of the hepatic parenchyma of fetus ‘A’, hemophagocytosis is indicated by an arrow (k); H&E. Mild and focal endocardial fibroelastosis in the heart of fetus ‘B’ on Masson’s trichrome (l) and elastica van Gieson (m) stains. Figure S3: Additional placental and cardiac histological findings. Myocardium of both fetus ‘A’ (a) and ‘B’ (b) appeared depleted and swollen on H&E staining. (c) Endocardial fibroelastosis was evident on Masson’s trichrome (upper) and elastica van Gieson (lower) stains. (d) Placental arteries showed irregular stenosis, without inflammatory infiltration; H&E. (e) Results of CD3 (upper) and CD8 (lower) immunohistochemistry. (f) Results of CD68 (upper) and CD71 (lower) immunohistochemistry.

## Abbreviations

The following abbreviations are used in this manuscript:

ANA	antinuclear antibody
AV	atrioventricular
B2GPI	beta-2 glycoprotein I
CHB	congenital heart block
DC	dichorionic
FcRn	neonatal Fc receptor
HCQ	hydroxychloroquine
H&E	hematoxylin and eosin
IFN	interferon
IHC	immunohistochemistry
IUFD	intrauterine fetal demise
IVIG	intravenous immunoglobulin
MC	monochorionic
UCTD	undifferentiated connective tissue disease
US	ultrasonography
