# Supplementary material for: Discordant Manifestation of Congenital Heart Block in a Twin Pregnancy: A Case Report
Source: Diagnostics (Basel). 2026 Sep 4;16(17):2846. doi: 10.3390/diagnostics16172846 (PMC13565159; doi:10.3390/diagnostics16172846)
Supplement: Supplementary file 1 [file diagnostics-16-02846-s001.zip › diagnostics-4471324-supplementary.pdf]

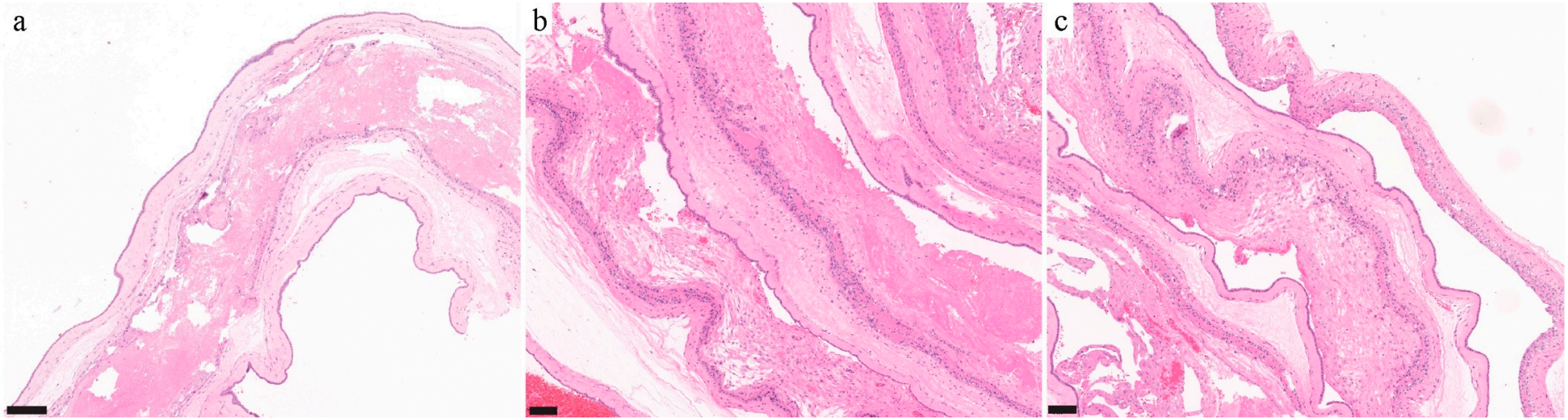

Figure S1. Fetal membranes. Dividing membrane (a) was consistent with dichorionic diamniotic placentation. Chorioamniotic membranes of fetus 'A' (b) and fetus 'B' (c); H&E.
Scale bar = 200 µm (a) and 100 µm (b-c).

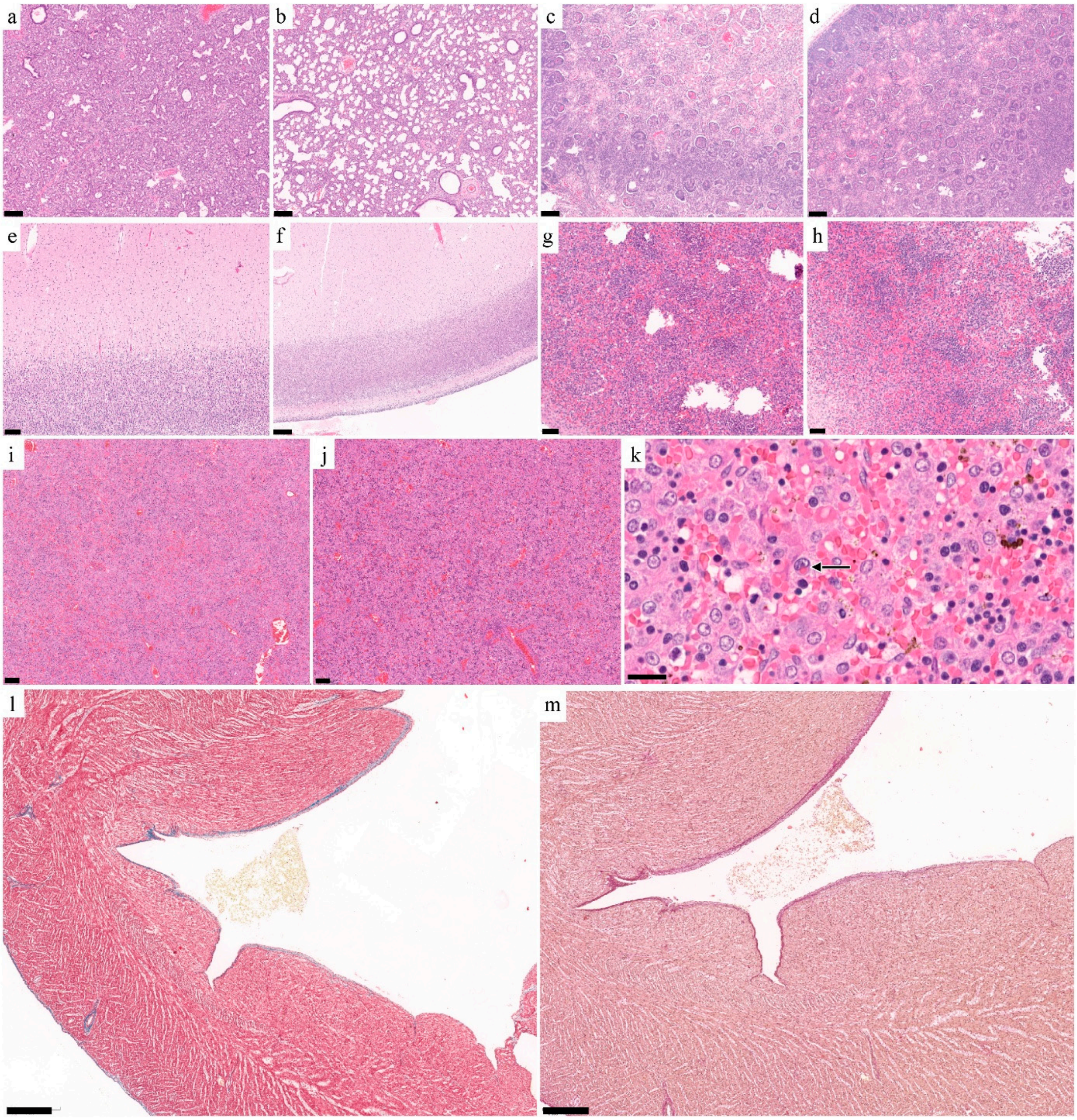

Figure S2. Additional histological findings. Pulmonary parenchyma of fetus ‘A’ (a) and ‘B’ (b). Renal parenchyma of fetus ‘A’ (c) and ‘B’ (d). Cerebral parenchmya of fetus ‘A’ (e) and ‘B’ (f). Splenic parenchyma of fetus ‘A’ (g) and ‘B’ (h). Hepatic parenchyma of fetus ‘A’ (i) and ‘B’ (j). High power magnification of the hepatic parenchyma of fetus ‘A’, hemophagocytosis is indicated by an arrow (k); H&E. Mild and focal endocardial fibroelastosis in the heart of fetus ‘B’ on Masson’s trichrome (l) and elastica van Gieson (m) stains.

Scale bar = 200 µm (a, b, g, h, k, m) and 100 µm (c, f, i, j)

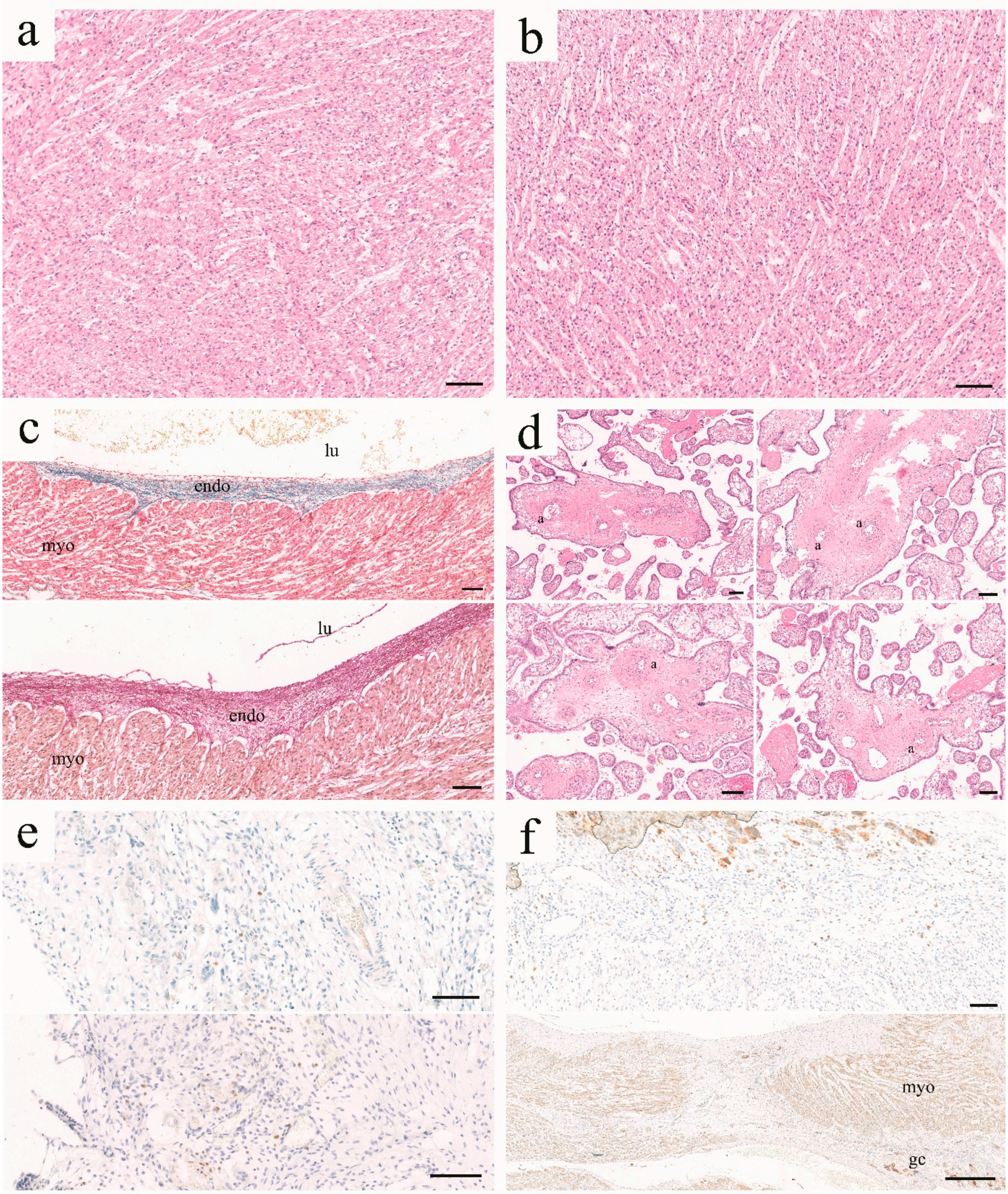

Figure S3. Additional placental and cardiac histological findings. Myocardium of both fetus 'A' (a) and 'B' (b) appeared depleted and swollen on H&E staining. (c) Endocardial fibroelastosis was evident on Masson's trichrome (upper) and elastica van Gieson (lower) stains. (d) Placental arteries showed irregular stenosis, without inflammatory infiltration; *H&E.* (e) Results of CD3 (upper) and CD8 (lower) immunohistochemistry. (f) Results of CD68 (upper) and CD71 (lower) immunohistochemistry.

Scale bar = 100 µm (a-e) and 500 µm (f); a: arteriole; endo: endocardium; gc: giant cell; lu: left ventricular lumen; myo: myocardium
